# Prevalence and Genetic Diversity of Enteric Viruses in Children with Diarrhea in Ouagadougou, Burkina Faso

**DOI:** 10.1371/journal.pone.0153652

**Published:** 2016-04-19

**Authors:** Nafissatou Ouédraogo, Jérôme Kaplon, Isidore Juste O. Bonkoungou, Alfred Sababénédjo Traoré, Pierre Pothier, Nicolas Barro, Katia Ambert- Balay

**Affiliations:** 1 Laboratoire de Biologie Moléculaire, d’Epidémiologie et de Surveillance des Bactéries et Virus Transmis par les Aliments, Centre de Recherche en Sciences Biologiques Alimentaires et Nutritionnelles (CRSBAN), Université de Ouagadougou, Ouagadougou, Burkina Faso; 2 National Reference Centre for enteric viruses, University Hospital of Dijon, Dijon, France; 3 Laboratoire National de Santé Publique, Direction de la Biologie médicale (DBM), Ouagadougou, Burkina Faso; Instituto de Higiene e Medicina Tropical, PORTUGAL

## Abstract

Enteric viruses are a major cause of diarrhea in children, especially those under five years old. Identifying the viral agents is critical to the development of effective preventive measures. This study aimed to determine the prevalence and genetic diversity of common enteric viruses in children under five years old in Burkina Faso. Stool samples from children with (n = 263) and without (n = 50) diarrhea disorders were collected in Ouagadougou, Burkina Faso from November 2011 to September 2012. Rotavirus, norovirus, sapovirus, astrovirus, adenovirus and *Aichivirus A* were detected using real-time or end-point (RT-)PCR. Rotavirus strains were G and P genotyped by multiplex RT-PCR and other viral strains were characterized by sequencing of viral subgenomic segements. At least one viral agent was detected in 85.6% and 72% of the symptomatic and asymptomatic patients, respectively. Rotavirus (63.5%), adenovirus (31.2%) and genogroup II norovirus (18.2%) were the most prevalent viruses in symptomatic patients, but only rotavirus and genogroup II norovirus were significantly associated with diarrhea (OR: 7.9, 95%CI: 3.7–17; OR: 3.5, 95%CI: 1–11.7, respectively). Sapovirus (10.3%), astrovirus (4.9%), genogroup I norovirus (2.7%) and *Aichivirus A* (0.8%) were less prevalent. The predominant genotype of rotavirus was G9P[8] (36.5%), and the predominant norovirus strain was GII.4 variant 2012 (71.4%). Among sapovirus, the genogroup II (87.5%) predominated. Astrovirus type 1 (41.7%) was the most frequent astrovirus identified. *Aichivirus A* belonged to the three genotypes (A, B and C). Enteric adenoviruses type 40 and 41 were identified in 10.2% and 5.1% respectively. Several cases of co-infections were detected. The results highlight the high prevalence and the high diversity of enteric viruses in Burkinabe children.

## Introduction

Gastroenteritis remains a major public health problem worldwide, especially among children. More than 700 million cases of acute gastroenteritis are estimated to occur annually in children less than 5 years old and the mortality associated with gastroenteritis has been estimated to 0.8 to 2 million per year [1]. Gastroenteritis can be due to infections (bacterial, fungal, parasitic or viral) or due to other causes such as food (dietary errors, dietary imbalance) or drugs (abuse of laxatives, antibiotics) [2, 3].

Recent advances in molecular diagnostic techniques have allowed recognizing viruses as major cause of diarrheal illnesses. In particular, rotaviruses (family *Reoviridae*) are the leading cause of severe diarrheal diseases and dehydration, which often lead to the hospitalization of infants and young children throughout the world [4–6]. Based on the VP6 gene sequence-based classification system, they are tentatively classified into nine groups (A-I) [7], with group A rotaviruses (RVA) being the most epidemiologically relevant in humans. The genotypes G and P of RVA correspond to VP7 (glycoprotein) and VP4 (protease sensitive protein), respectively. RVA are classified into at least 27 G and 37 P genotypes [8]. The common human RVA genotypes that are circulating worldwide include: G1P[8], G2P[4], G3P[8], G4P[8] and G9P[8] [6, 9].

Noroviruses (NoV, family *Caliciviridae*) are the second most common cause of viral diarrhea in children less than 5 years old [10]. Noroviruses are genetically diverse and are divided into 7 genogroups (GI–GVII) and more than 30 genotypes according to comparison of VP1 sequences [11]. Only 3 genogroups (GI, GII and GIV) infect humans. Although several NoV strains circulate GII.4 is the predominant genotype infecting humans [10].

Other viral agents, including enteric adenoviruses type 40 and 41 (AdV, family *Adenoviridae*), human astroviruses (AstV, family *Astroviridae*), *Aichiviruses A* (AiV, family *Picornaviridae*) and human sapoviruses (SaV, family *Caliciviridae*) are also implicated as major causes of sporadic cases and outbreaks of childhood diarrhea [12–14].

In most sub-Saharan African countries including Burkina Faso, microbiological methods for investigation of diarrheal diseases are usually restricted to conventional enteric bacteria identification (such as *Salmonella sp*, *Shigella sp* and Enteropathogenic *Escherichia coli*) and viral etiology is rarely investigated. Previous studies showed that RVA was a common cause of childhood diarrhea in Burkina Faso [15–19] and subsequently RVA vaccine (RotaTeq^®^, Merck and Co, Whitehouse Station, NJ, USA) was introduced into the national immunization program in October 2013. Recently NoV and SaV were detected in cases of childhood diarrhea in Burkina-Faso [20–22]. However, the contribution of other enteric viruses such as AdV, AstV and AiV in cases of diarrhea in Burkina Faso remains unknown. It is therefore essential to fill the knowledge gap on these pathogens to be able to planify control strategies of diarrheal disease in the country. The aim of this study was to determine the prevalence and the molecular characteristics of these enteric viruses in Burkinabe children suffering from diarrhea diseases.

## Materials and Methods

### Ethics statement

Parents of the pediatric patients were informed on the study details and their oral consent was obtained and documented in the questionnaire form before stool specimen collection. In Burkina Faso, oral consent is sufficient for patients coming to hospital routinely for gastroenteritis and not because of the study, according to guidelines from the ministry of health. Written consent was obtained and documented in a consent form from guardians of the control group. The study protocol and consent procedure was approved by the Ethics Committee of Burkina Faso and the ministry of health.

### Sample collection and viral RNA/DNA extraction

This study was based on a relative low socio-economic status urban population of Ouagadougou (capital of Burkina Faso) consulting at Peripheral Health Centers.

Between November 2011 and September 2012, stool specimens were collected from 263 children under 5 years of age consulting for treatment of gastroenteritis in three hospitals in Ouagadougou: District hospital of Bogodogo (61 children), pediatric clinic “les Tissérins” (128 children) and Medical center with surgical antenna “Paul VI” (74 children). Diarrhea was defined according to the WHO criteria for children as the occurrence of three or more loose, liquid, or watery stools within a 24-hour period. Control stool samples were collected from 50 randomly selected children under five years old coming to the same health centers at the same period for routine immunization and not presenting gastroenteritis/diarrhea symptoms. One stool sample was collected from each patient and stored at -20°C until analysis. Viral nucleic acids were extracted from 20% fecal suspensions in phosphate-buffered saline using the NucliSENS^®^ EasyMAG^TM^ platform (bioMérieux, Marcy l’Etoile, France) according to the manufacturer’s instructions.

### Viral detection

RVA, NoV-GI, NoV-GII, SaV, and AstV were screened by real-time reverse transcription PCR (RT-PCR) using the TaqMan^®^ Fast Virus One-Step Master Mix on a 7500 Real-Time PCR system (Applied Biosystems, Foster City, CA, USA) with primers and probes described previously [23–27] (Table 1). AdV were detected using a commercial real-time PCR assay designed to detect all types of human AdV (Adenovirus R-gene^TM^, Argène, Verniolle, France). AiV were detected by end-point RT-PCR using the Qiagen One-Step RT-PCR kit (Qiagen, Hilden, Germany) and the primer set Ai6261/Ai6779 [28].

**Table 1:** Primer and probes used for viral detection.

	Primer or probe	Sequence (5’to 3’)	Polarity	References
	VP2-F1	TCTGCAGACAGTTGAACCTATTAA	+	
**Rotavirus**	VP2-F2^a^	CAGACACGGTTGAACCCATTAA	+	[23]
	VP2-F3^a^	TCGGCTGATACAGTAGAACCTATAAATG	+	
	VP2-F4^a^	TGTCAGCTGATACAGTAGAACCTATAAATG	+	
	VP2-F5^a^	TCAGCTGACACAGTAGAACCTATAAATG	+	
	VP2-R1^a^	GTTGGCGTTTACAGTTCGTTCAT	-	
	VP2-R2^a^	GTTGGCGTCTACAATTCGTTCAT	-	
	VP2-P^b^	6FAM-ATGCGCATRTTRTCAAAHGCAA	++	
**Norovirus Génogroupe I**	JJV1NF ^a^:	CCA TGT TCC GTT GGA TGC	+	[24]
	JJV1R ^a^:	TCC TTA GAC GCC ATC ATC AT	-	
	JJV1P ^b^:	FAM-5’-TGT GGA CAG GAG ATC GCA ATC TC -3’-TAMRA	+	
	RING-1b ^b^ :	FAM-5’-AGA TCG CGG TCT CCT GTC CA-3’-TAMRA	-	
**Norovirus Génogroupe II**	QNIF2d ^a^ :	ATG TTC AGR TGG ATG AGR TTC TCW GA	+	[25]
	COG2R^a^ :	TCG ACG CCA TCT TCA TTC ACA	-	
	QNIFS ^b^ :	FAM-5’-AGC ACG TGG GAG GGC GAT CG -3’-TAMRA	+	
**Astrovirus**	AV1TR^a^	CCG AGT AGG ATC GAG GGT	-	[26]
	AV2TR^a^	GCT TCT GAT TAA ATC AAT TTT AA	+	
	AVs^b^	FAM-5'-CTT TTC TGT CTC TGT TTA GAT TAT TTT AAT CAC C-3'-TAMRA		
**Sapovirus**	SaV124F^a^	GAY CAS GCT CTC GCY ACC TAC	+	[27]
	SaV1Fa^a^	TTG GCC CTC GCC ACC TAC	+	
	SaV1245R^b^	CCC TCC ATY TCA AAC ACT A	-	
	SaV124TP^b^	FAM-CCR CCT ATR AAC CA-MGB	-	
***Aichivirus A***	AICHI 6261^a^	ACA CTC CCA CCT CCC GCC AGT A	+	[28]
	AICHI 6779^a^	GGA AGA GCT GGG TGT CAA GA	-	

^a^ Primer

^b^ Probe

### Genotyping of viral strains

All positive samples by real-time (RT-)PCR were characterized by end-point (RT-)PCR. RVA positive samples were G and P genotyped using multiplex RT-PCR according to the EuroRotaNet methods (www.eurorota.net/docs.php). Caliciviruses were genotyped using primer sets SR80/NVP110 [29] and JV12/JV13 [30] to amplify a fragment of the RNA polymerase genes of SaV and NoV, respectively. Primer sets G1SKF/G1SKR and G2SKF/G2SKR [31] were used to amplify a fragment of the capsid genes of NoV-GI and NoV-GII, respectively. AstV positive samples were typed by sequencing Mon244 and Mon245 [32] RT-PCR products. All RT-PCRs were performed using the Qiagen OneStep RT-PCR kit (Qiagen, Hilden, Germany) according to the manufacturer’s instructions. AdV positive samples were typed by PCR using the AmpliTaq^®^ DNA Polymerasewith Buffer II (Applied Biosystems, Foster City, CA, USA) and the primer set Hex1DEG/Hex2DEG followed by nested PCR with primers Hex3DEG/Hex4DEG [33].

Genotyping of NoV (GI and GII), SaV, AstV, AdV and AiV was performed by direct sequencing of the PCR products with the same primers used for amplification by using an ABI PRISM^®^ BigDye^®^ Terminator Cycle Sequencing Kit on a 3130XL DNA Genetic Analyzer (Applied Biosystems, Foster City, CA, USA). Sequencing of the PCR amplicons for the VP7 gene RVA of unknown G types and VP4 gene RVA of unknown P types was conducted with primers VP7-F/VP7-R and VP4-F/VP4-R respectively [34].

### Sequence analysis

Viral sequences were compared with reference strains available in the European Nucleotide Archive (ENA) database using the FASTA program from the European Bioinformatics Institute (www.ebi.ac.uk). For norovirus, genotyping was also determined using the Norovirus Genotyping Tool version 1.0 [35].

Phylogenetic analyses were performed with the Molecular Evolutionary Genetics Analysis (MEGA) software version 6.0 software packages [36]. Phylogenetic trees were constructed by the Maximum Likelihood method with the Kimura 2-parameter as substitution model, and including the reference sequences available on the GenBank. The confidence values of the internal nodes were calculated by performing 1000 bootstrap replicates. Phylogenetic analysis of the amino-acids and nucleotides alignments was performed by using pairwise distance methods

The nucleotide sequences of amplicons of Burkinabe strains were submitted to the ENA database under the following accession numbers: RVA G6 and G12 detected (VP7 gene, 842 bp, LN612503 to LN612530), NoV-GI (ORF 2, 302 bp, LN612531 to LN612535 and ORF 1, 285 bp, LN612550 to LN612552), NoV-GII (ORF 2, 302 bp LN612536 to LN612549 and ORF 1, 285 bp LN612553 to LN612571), SaV (ORF 1, 277 bp, LN612572 to LN612579), AstV (ORF 2, 373 bp, LN612580 to LN612591), AiV (3CD, 477 bp, LN612592 to LN612595) and AdV types 40/41 (Hexon protein gene, 255 bp, LN612596 to LN612604).

### Statistical analysis

Statistical analyses were performed with Stata^®^ software (StataCorp release 10, 2007; College Station, TX, USA). We compared categorical data by the Chi-square and Fischer exact tests when the population was less than 5. The level of confidence was 95% for all confidence intervals (CI). The comparison of detection of viruses between the group of children with diarrhea and the control group was evaluated as an odds ratio (OR). A *p*-value less than 0.05 were considered statistically significant.

## Results

### Detection of enteric viruses in stools

The mean age of children less than five years old with symptoms of diarrhea was 14.1 months (versus 11.2 months in control group) and the median age was 10 months (versus 6 months in control group). The sex ratio (male: female) was 1.1 for the children with diarrhea versus 0.7 for the children without diarrhea.

Among the 263 stool specimens from patients with diarrhea symptoms, 225 (85.6%) contained at least one virus, versus 36 (72%) in the control group. Table 2 summarizes the number of viruses found in children with diarrhea and children without diarrhea. RVA was the most prevalent virus in symptomatic children (63.5%) and has the strongest association with disease [p<0.001; OR: 7.9, 95%CI: 3.7–17]. AdV was the second most prevalent virus in symptomatic children (31.2%), but the presence of this virus was statistically higher in the control group than in the symptomatic patients (p = 0.01). With a prevalence of 20.9%, NoV was the third most prevalent virus in symptomatic children. Among NoV, NoV-GII was the most frequently detected and was associated with disease (p = 0.04, OR = 3.5). NoV-GI, SaV, AstV and AiV circulated at lower frequencies and their prevalence in the symptomatic group was not significantly different than in the control group (p = 0.5, p = 0.4, p = 0.4 and p = 0.09, respectively).

**Table 2:** Prevalence of viruses in children with diarrhea and children without diarrhea.

	No. positive (%)			
**Virus**	Children’s with diarrhea disorder (n = 263)	Children’s without diarrhea disorders (n = 50)	Odds Ratio (95% CI)^a^	P value
**Rotavirus**	167 (63.5%)	9 (18%)	7.9 (3.7–17)	<0. 0001
**Norovirus**	55 (20.9%)	3 (6%)	4.1 (1.2–13. 8)	0. 02
**Norovirus GI**	7 (2.7%)	0 (0%)	2.9 (0.2–52.5)	0.5
**Norovirus GII**	48 (18.3%)	3 (6%)	3.5 (1–11.7)	0. 04
**Sapovirus**	27 (10.3%)	3 (6%)	1.8 (0.5–6.2)	0.4
**Astrovirus**	13 (4.9%)	1 (2%)	2.5 (0.3–19.9)	0.4
**Adenovirus**	82 (31.2%)	25 (50%)	0.5 (0.2–0.8)	0. 01
***Aichivirus A***	2 (0.8%)	2 (4%)	0.2 (0.02–1.3)	0.09

^a^ CI, Confidence Interval

The distribution of positive samples in children with diarrhea disorders according to age, gender and hospitalization status is shown in Table 3. No significant difference was observed according to age, gender and hospitalization status (p>0.05, data not shown).

**Table 3:** Distribution of positive samples in children with diarrhea disorders according to age, gender and hospitalization status.

	Rotavirus	Norovirus GI	Norovirus GII	Sapovirus	Adenovirus	Astrovirus	*Aichivirus A*
	(n = 167)	(n = 7)	(n = 48)	(n = 27)	(n = 82)	(n = 13)	(n = 2)
**Age (months)**							
0–12	87(70.2%)	2 (1.6%)	19 (15.3%)	11(8.9%)	41(33.1%)	8 (6.5%)	1 (0.8%)
n = 124							
13–24	50 (59.5%)	4 (4.8%)	21(25%)	13 (15.5%)	25(29.8%)	4 (4.8%)	1(1.2%)
n = 84							
25–59	30 (50.8%)	1 (1.8%)	8 (14.5%)	3(5.5%)	16(29.1%)	1 (1.8%)	0
n = 55							
**Gender**							
Female	92 (55%)	6 (85.7%)	18(37.5%)	13(48.1%)	48 (58.5%)	5(38.5%)	2(100%)
Male	75 (45%)	1(14.3%)	30(62.5%)	14(51.9%)	34 (41.5%)	8(61.5%)	0
**Hospitalized/Non-hospitalized**							
Hospitalized	48 (29%)	3 (42.8%)	14 (29.2%)	5 (18.5%)	21 (25.6%)	3 (23.1%)	2 (100%)
Non-Hospitalized	119 (71%)	4 (57.1%)	34 (70.8%)	22 (81.5%)	61 (74.4%)	10 (76.9%)	0

Enteric viruses were detected throughout the study period in samples from children suffering for diarrhea with a peak of infection observed for RVA, AdV and NoV during the cold season (November to February) (data not shown).

Mixed infections were found in 94 cases (35.7%) from children suffering for diarrhea (Table 4) and only two cases (4%) in control group ((AdV+AstV+ SaV and AdV+AiV). The most common mixed infections in the group with diarrhea were RVA combined with one other virus, notably AdV (45.7%) or NoV GII (18.1%). Of note, a maximum of four different viruses were simultaneously detected in stool samples from two patients with diarrhea.

**Table 4:** Pattern of coinfections of enteric viruses.

Coinfections pattern	Number (%)
AdV + SaV	3 (3.2)
RVA + AdV	43 (45.7)
RVA + AdV + SaV	5 (5.3)
RVA + SaV	7 (7.4)
RVA + NoV-GI	1 (1.1)
RVA + NoV-GII	17 (18.1)
RVA + NoV-GI + SaV	1 (1.1)
RVA + NoV-GII + SaV	2 (2.1)
RVA + NoV-GII + SaV + AstV	1 (1.1)
RVA + NoV-GII + AdV	2 (2.1)
RVA + NoV-GI + NoV-GII + AdV	1 (1.1)
NoV-GI + AdV	2 (2.1)
NoV-GII + AdV	7 (7.4)
NoV-GII + SaV	1 (1.1)
NoV-GI + NoV-GII + SaV	1 (1.1)

### Molecular epidemiology of viral infections

A relatively large proportion of “untyped sample” was found in this study. All the “untyped samples” corresponded to samples that failed to be amplified by genotyping end-point (RT-)PCR. This was due to low viral load. As seen in Table 5, Ct-values were significantly higher for the viruses that could not be genotyped, as compared to viruses that could be genotyped.

**Table 5:** Comparison between mean Ct-values of genotyped viruses typed and mean Ct-values of untyped viruses.

	Ct values mean (+/- Sd*)		
	Typed	Untyped	p value
**Rotavirus**	16.00 (+/- 6.6)	33.85 (+/- 3.9)	< 0 .001
**Norovirus**	21.1 (+/- 5.8)	30.2 (+/- 2.8)	< 0 .001
**Sapovirus**	18.08 (+/-3.5)	30.85 (+/- 5.9).	< 0 .001
**Astrovirus**	26.61(+/- 6.9)	37.27 (+/- 0.5)	0.05
**Adenovirus**	29.54 (+/- 7.8)	35.88 (+/- 3.3)	< 0 .001

*Sd: Standard deviation

#### Rotaviruses

**Fig 1:**
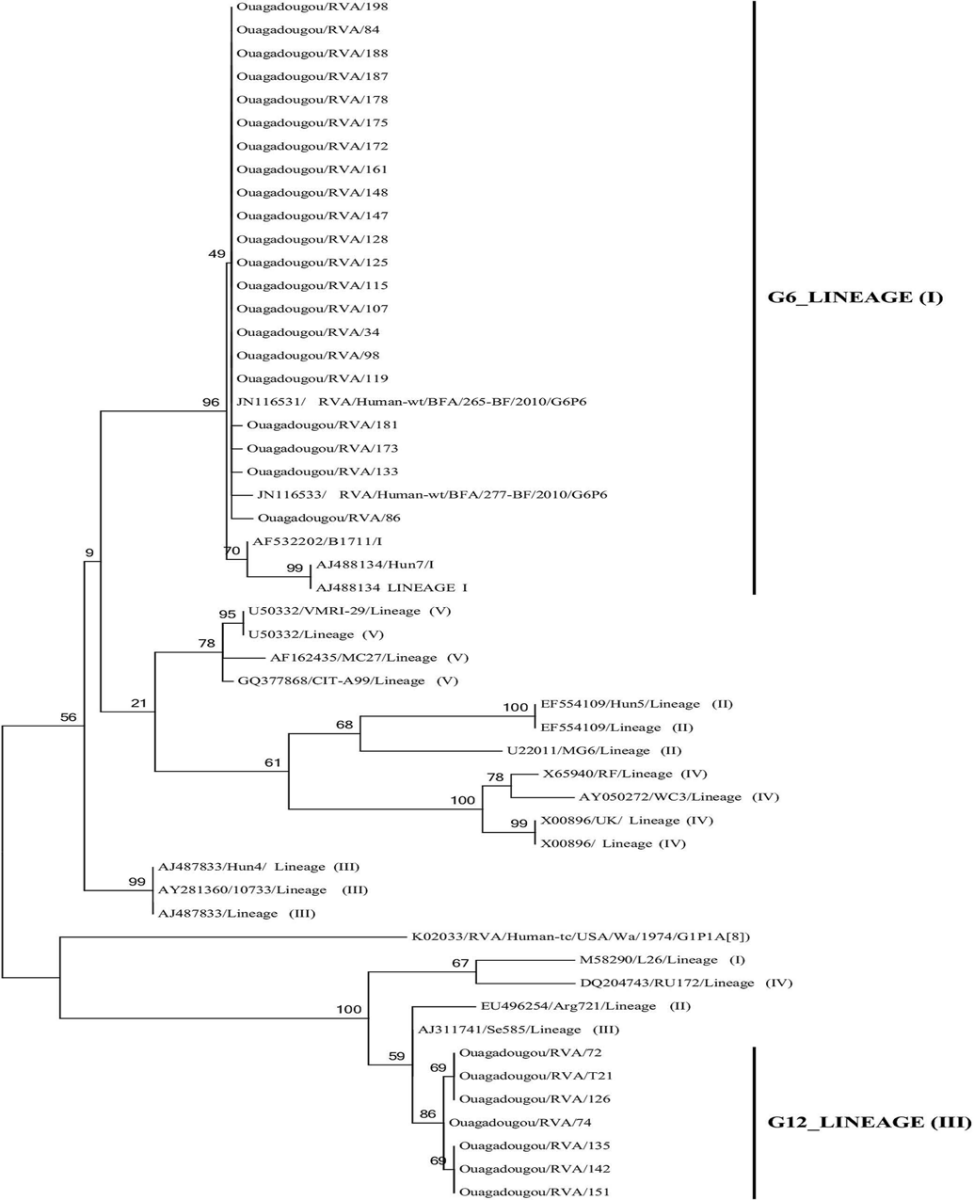
Phylogenetic analysis based on the partial nucleotide sequences (842 bp) of the rotavirus VP7 gene detected in Ouagadougou, Burkina Faso, between November 2011 and September 2012. The tree was constructed using Maximum likelihood clustering. Reference strains of rotavirus were selected from the GenBank database.

The distribution of G and P types is shown in Table 6. Among the positive rotaviruses (167 in symptomatic group and 9 in control group), both G and P genotypes could be assigned for 54.5% (n = 96), only P genotype for 5.1% (n = 9), only G genotype for 9.7% (n = 17) and both G and P genotypes remained undetermined for 30.7% (n = 54). Among the 122 samples successfully genotyped (even partially), rotavirus genotypes G9 (45.9%), G6 (17.2%) and G1 (13.1%) predominated. Genotypes G3 (6.6%), G12 (5.7%) and G2 (4.1%) were detected at lower frequencies. Regarding the P genotypes, P[8] (59%) predominated, followed by P[6] (24.6%) and P[4] (1.6%). Among the completely characterized strains, a wide variety of rotavirus combinations was observed, with G9P[8] (36.5%) predominating, followed by G6P[6] (16.7%), G1P[8] (14.6%) and G9P[6] (10.4%) G12P[8] (6.3%), G3P[8] (6.3%), G2P[8] (3.1%), G3P[6] (2.1%), G1P[4] (1%), G2P[6] (1%), G9P[4] (1%) and G9P[4]+P[8] (1%) were detected at lower frequencies. Phylogenetic tree based on the partial nucleotide sequences (842 bp) of the VP7 gene is showed in Fig 1. The G12 strains found in this study displayed the highest sequence identities with lineage III strains (98.1–98.3% nucleotide (nt) and 98.2–99.1% amino-acid (aa) identities with the reference strain RVA/Human-wt/USA/Se585/1999/G12P[6], Accession number AJ311741) and the G6 strains clustered within lineage I (95.5–96.8% nt and 96.9–97.9% aa identities with the reference strain RVA/Human-wt/HUN/Hun7/1998/G6P[9] (AJ488134). All G6 were strongly related to previously RVA G6 found in Burkina Faso (RVA/Human-wt/BFA/265-BF/2010/G6P6 (JN116531)) with 100% nt (100% aa) identity.

**Table 6:** Distribution of rotavirus G and P genotypes among Children under 5 years of age in Burkina Faso, November 2011-September 2012.

P Type	G Type							
	G1	G2	G3	G6	G9	G12	G-UD	All
P[4]	1	0	0	0	1	0	0	2
P[6]	0	1	2	16	10	0	1	30
P[8]	14	3	6	0	35	6	8	72
P[4]+P[8]	0	0	0	0	1	0	0	1
P-UD	1	1	0	5	9	1	54	71
All	16	5	8	21	56	7	63	176

UD, UnDetermined

#### Noroviruses GI

**Fig 2:**
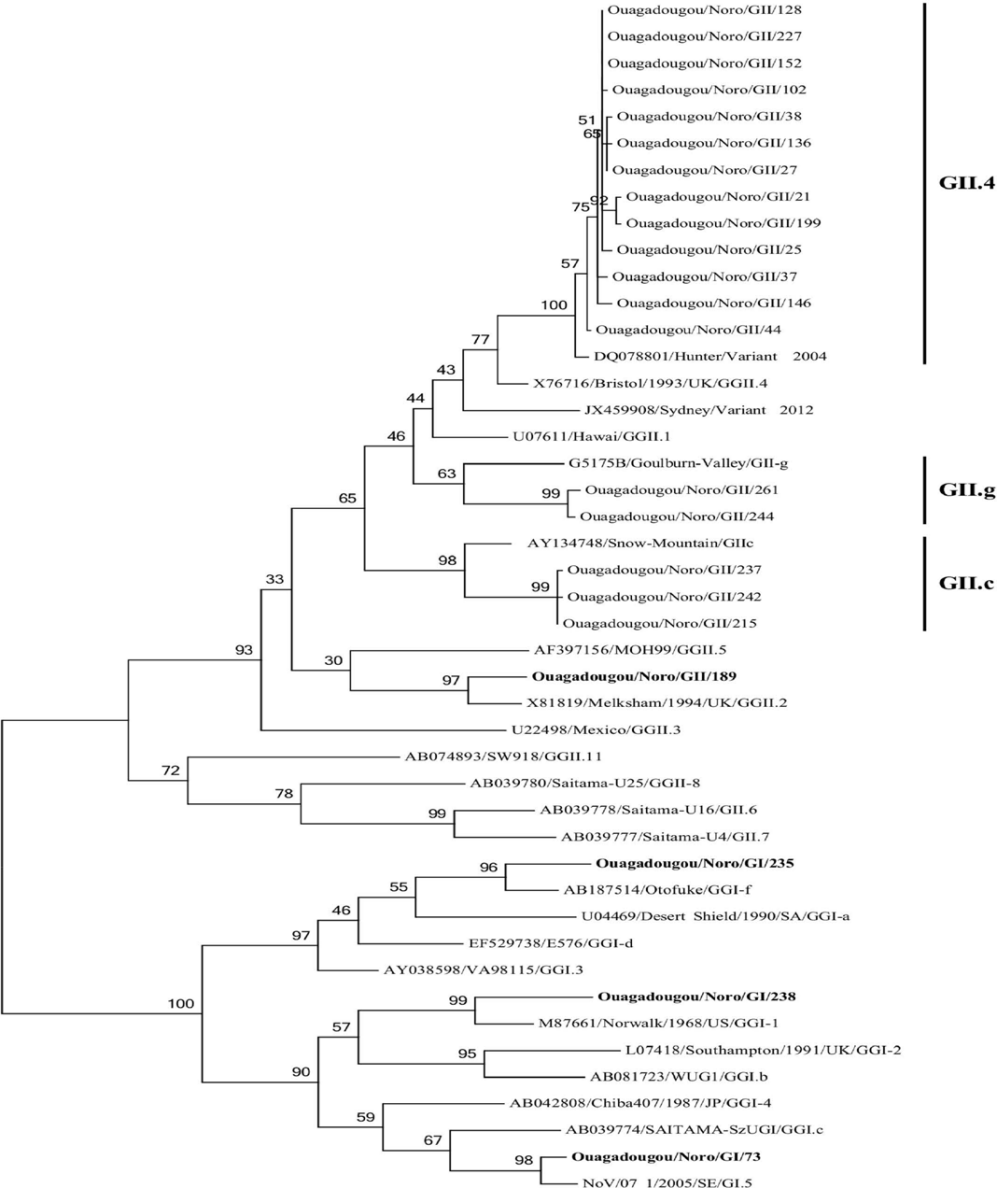
Phylogenetic analysis based on the partial nucleotide sequences (285 bp) of the RNA-dependent RNA polymerase coding gene of the norovirus strains detected in Ouagadougou, Burkina Faso, between November 2011 and September 2012. The tree was constructed using Maximum Likelihood method. Reference strains of norovirus were selected from the GenBank database.

**Fig 3:**
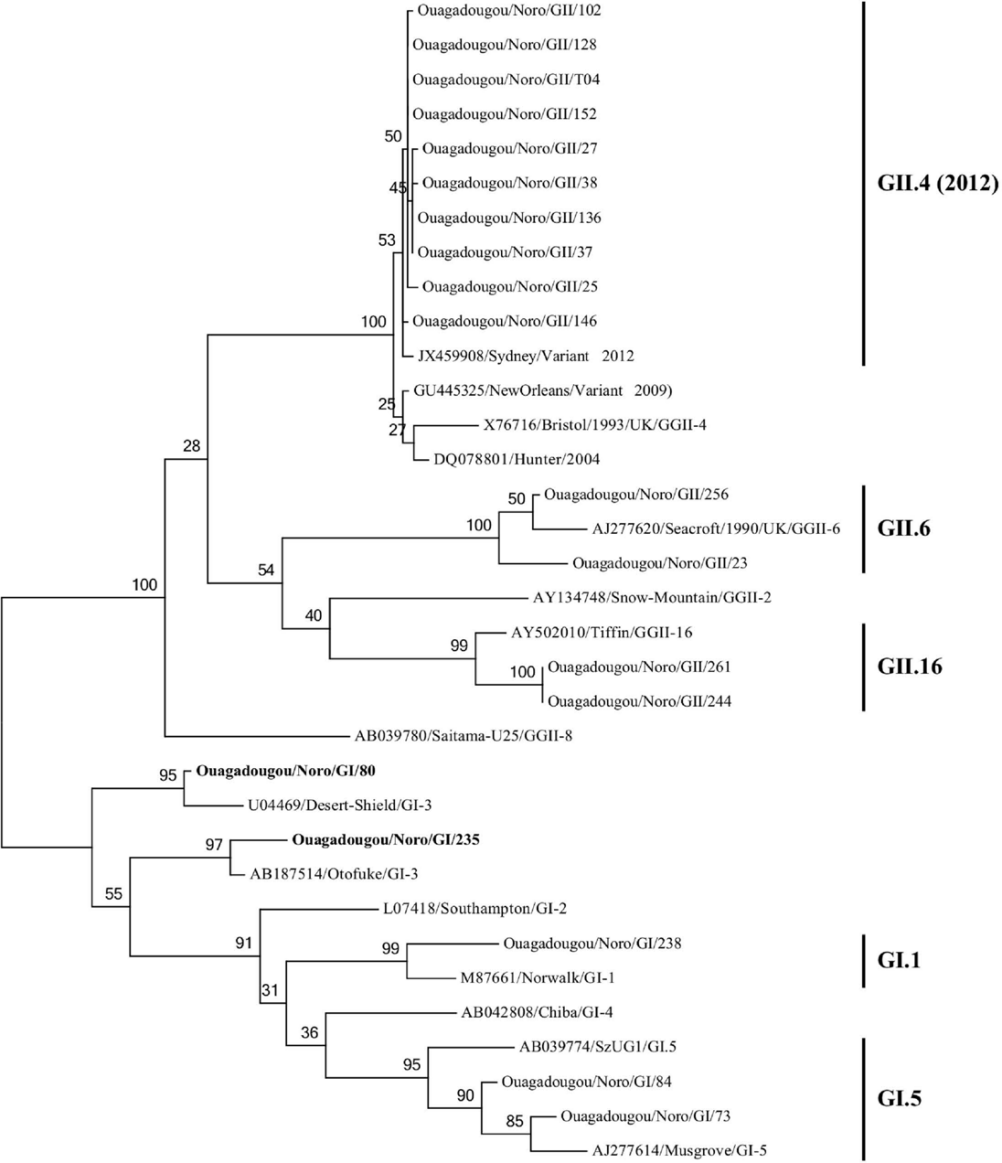
Phylogenetic analysis based on the partial nucleotide sequences (302 bp) of the capsid coding gene of NoV strains detected in Ouagadougou, Burkina Faso, between November 2011 and September 2012. The tree was constructed using Maximum Likelihood method. Reference strains of norovirus were selected from the GenBank database.

Five NoV-GI strains out of 7 were successfully genotyped: three strains were genotyped based on both the RNA polymerase and the capsid sequences (GI.1, n = 1; GI.f/I.3, n = 1; GI.5, n = 1) and two only on the capsid sequence (GI.3, n = 1; GI.5, n = 1). Based on the nucleotide sequences, representative phylogenetic trees of the polymerase and capsid regions are showed in Figs 2 and 3 respectively. Phylogenetic analysis showed that the GGI.1 strain identified in this study was closely related to the Norwalk (Accession number, M87661) (96.8 and 90% identity respectively aa and nt), GGI.3 were related to Desert Shield (Accession number, U04469) with 98.9% aa and 95.2nt, and GGI.5 were related to MUSGROVE (Accession number, AJ277614) with 98.9% aa and 91.3–93.4% nt.

#### Noroviruses GII

Twenty four NoV-GII strains out of 51 were successfully genotyped: ten strains were genotyped based on both the RNA polymerase and the capsid sequences (GII.4, n = 10), four only on the capsid sequence (GII.16, n = 2; GII.6, n = 2) and ten only on the RNA polymerase sequence (GII.4, n = 4; GII.2, n = 1; GII.c, n = 3; GII not assigned, n = 2). Phylogenetic tree based on the polymerase and capsid regions are showed in Figs 2 and 3. Phylogenetic analysis showed that the GGII.4 strains presented 97.9% amino-acids identity (93–94.7% nt identity) in the capsid region and 94.7–95.7% amino-acids identity (88.8–90% nt identity) in the polymerase region with the reference strain Bristol/1993/UK (X76716). Also GGII.4 strains were closely related to the GGII.4 variant 2012 (Sydney) (JX459908) for the capsid (99% nt and 100% aa) and related to the GGII.4 variant 2004 (Hunter) (DQ078801) for the RNA polymerase region (97% nt and 100% aa). The GII.c related with the reference strain Snow-Mountain (AY134748) (90% nt and 100% aa). The GGII.g related with the reference strain Goulburn-Valley (G5175B) (90% nt and 97% aa).

#### Sapoviruses

**Fig 4:**
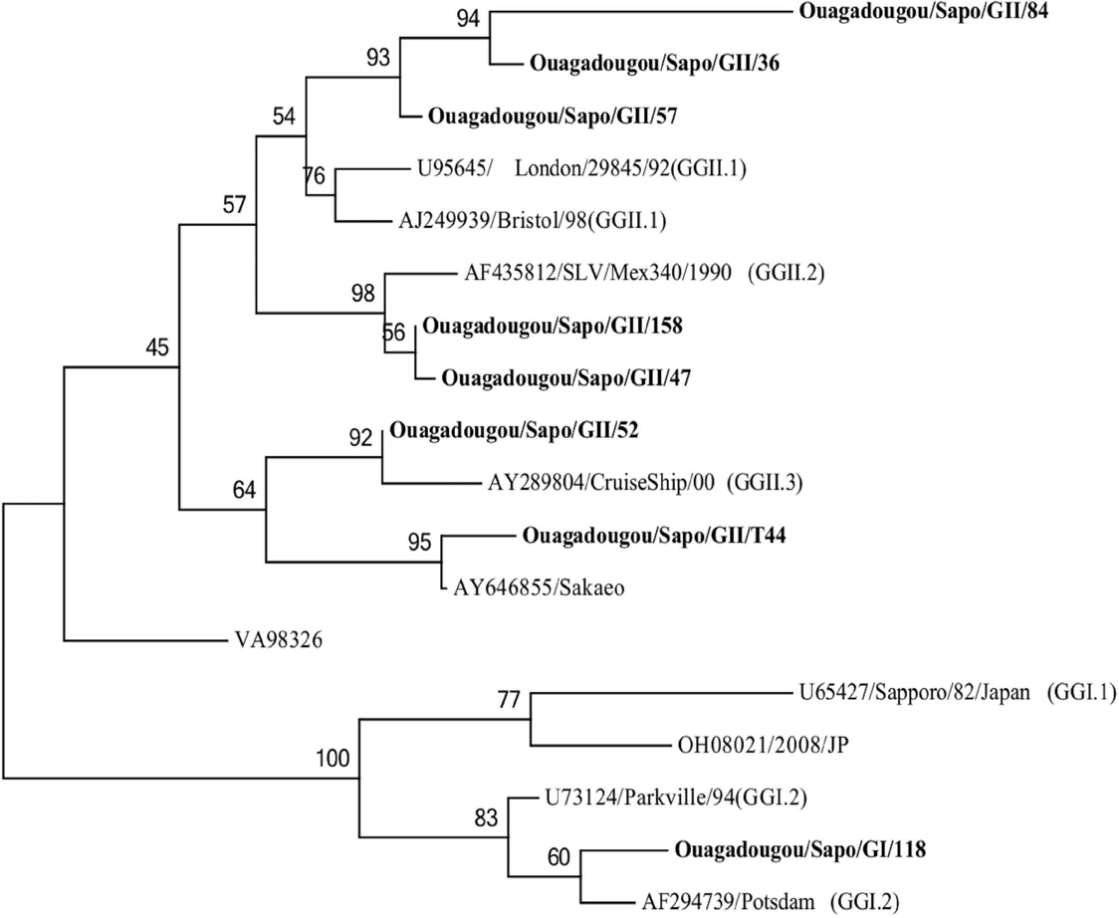
Phylogenetic analysis based on the partial nucleotide sequences (277 bp) of an area located in the open-reading frame (ORF2) of sapovirus strains detected in Ouagadougou, Burkina Faso, between November 2011 and September 2012. The tree was constructed using Maximum Likelihood method. Reference strains of sapovirus were selected from the GenBank database.

Phylogenetic tree based on the partial nucleotide sequences (277 bp) of an area located in the open-reading frame (ORF2) of sapovirus is showed in Fig 4. Eight SaV strains out of 30 were successfully genotyped and fell into two distinct genogroups: GI (1/8; 12.5%) and GII (7/8; 87.5%). GII strains were further classified into genotypes GII.1 (n = 3), GII.2 (n = 2) and GII.3 (n = 1) and in a new cluster of GII (n = 1) represented by the reference strain SaKaeo-15 (AY646855). The GI strain was classified into GI.2. Phylogenetic analysis showed that the GII.1 strains presented 72–89% nucleotides identity (60–98% aa identity) with the reference strain Bristol/98 (AJ249939). The GII.2 presented 93% nucleotide identity (99% aa identity) with the reference strain SLV/Mex340/1990 (AF435812). The new cluster of GII related to the reference strain SaKaeo-15 (AY646855) with 93% nucleotide (99% amino-acids). The GII.3 displayed 94% nucleotides identity (99% aa identity) with the reference strains CruiseShip/00 (AY289804). GI.2 strain shared 93% nucleotide (100% aa) with the reference strain Potsdam/GI.2 (AF294739).

#### Adenoviruses

**Fig 5:**
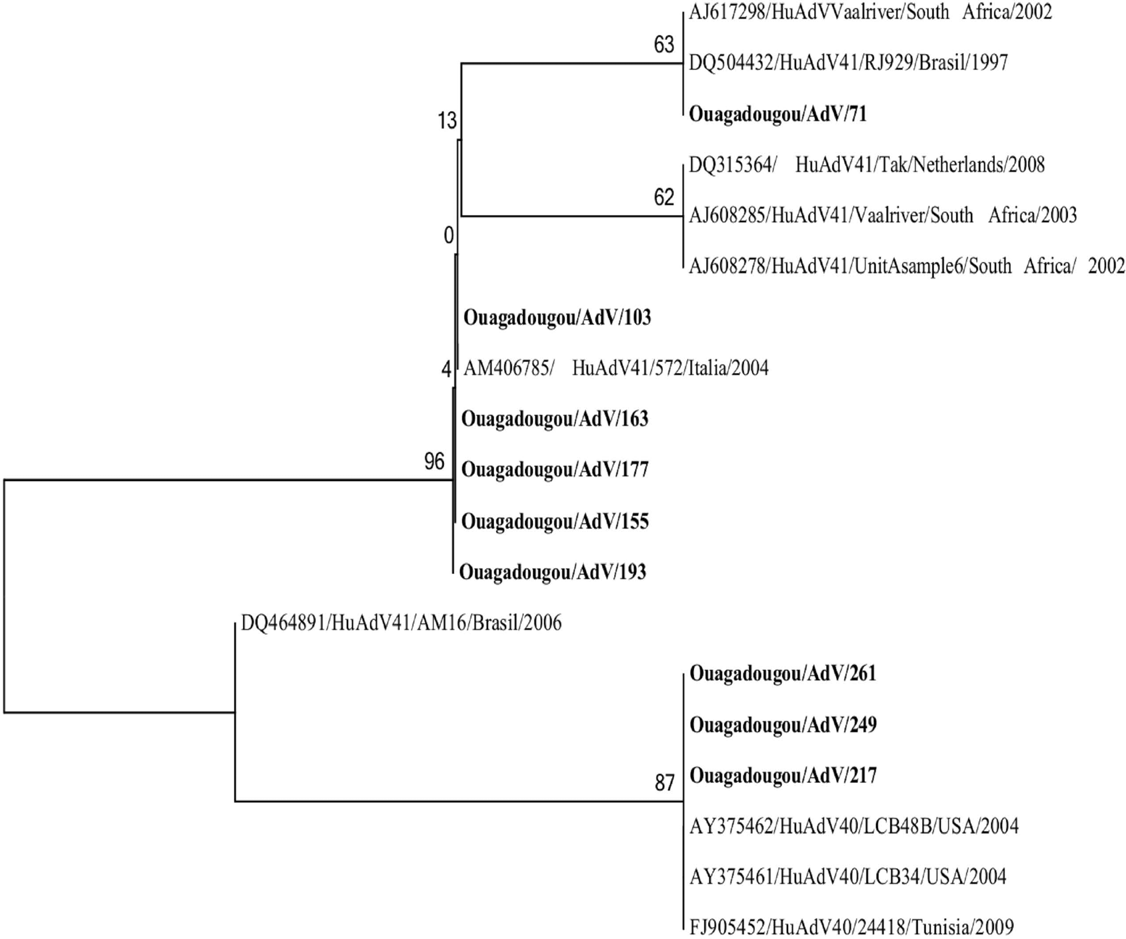
Phylogenetic analysis based on hexon protein gene of AdV 40/41 of human adenovirus strains detected in Ouagadougou, Burkina Faso, between November 2011 and September 2012. The tree was constructed using Maximum Likelihood method. Reference strains of adenovirus type 40 and type 41 were selected from the GenBank database.

Fifty nine adenovirus strains out of 107 were successfully genotyped. Enteric adenoviruses were identified in 12/59 samples (20.3%), with type 41 detected in 6/59 (10.2%), type 40 detected in 3/59 (5.1%) and type 31 detected in 3/59 (5.1%). Phylogenetic tree based on hexon protein gene of AdV 40/41 is showed in Fig 5. AdV type 40 strains related to the reference strain HuAdV40/24418 (FJ905452) with 100% nt identity (100% aa) and Adv type 41 displayed to the reference strain HuAdv41/UnitAsample6/South Africa/ 2002 (AJ608278) with 94–100% nt identity (97% aa). Others AdV were type 6 (16/59; 27.1%), type 2 (6/59; 10.2%), type 1 (1/59; 1.7%), type 12 (1/59; 1.7%), type 13 (1/59; 1.7%), type 18 (1/59; 1.7%), type 23 (1/ 59; 1.7%), type 3 (1/59; 1.7%), type 37 (3/59; 5.1%), type 5 (3/59; 5.1%), type 7(2/59; 3.4%), type 9 (1/59; 1.7%) and type D (10/59; 16.9%).

#### Astroviruses

**Fig 6:**
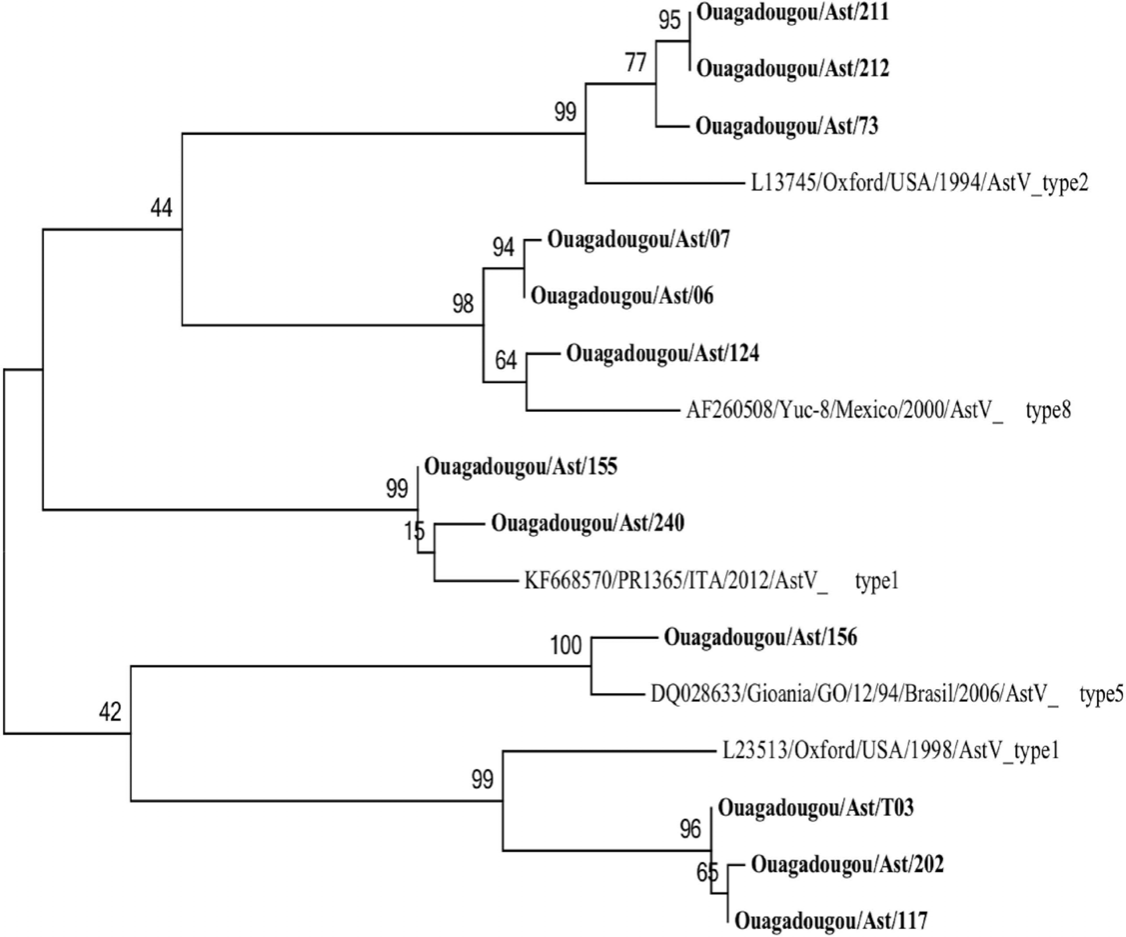
Phylogenetic analysis based on the partial nucleotide sequences (373 bp) of an area located in the open-reading frame (ORF2) of Astrovirus detected in Ouagadougou, Burkina Faso, between November 2011 and September 2012. The tree was constructed using Maximum Likelihood method. Reference strains of astrovirus were selected from the GenBank database.

Phylogenetic tree based on the partial nucleotide sequences (373 bp) of an area located in the open-reading frame (ORF2) of astrovirus is showed in Fig 6. In this analysis, 5 out of 12 strains of Astrovirus (41.67%) were characterized as type 1 (AstV-1). Three strains (25%) were characterized as type 2 (AstV-2), one (8.33%) as type 5 (AstV-5) and three (25%) as type 8 (AstV-8). All Ast type 1 presented 78–91% nt identity (97–100% aa) with the reference strain Oxford/Type 1 (L23513), but two strain of Ast type 1 found in this study were strongly related with the reference strain ITA/2012/PR1365 (KF668570). Ast type 2 presented 94% nt identity (100% aa) with the reference strain Oxford/type 2 (L13745).

#### Aichiviruses A

**Fig 7:**
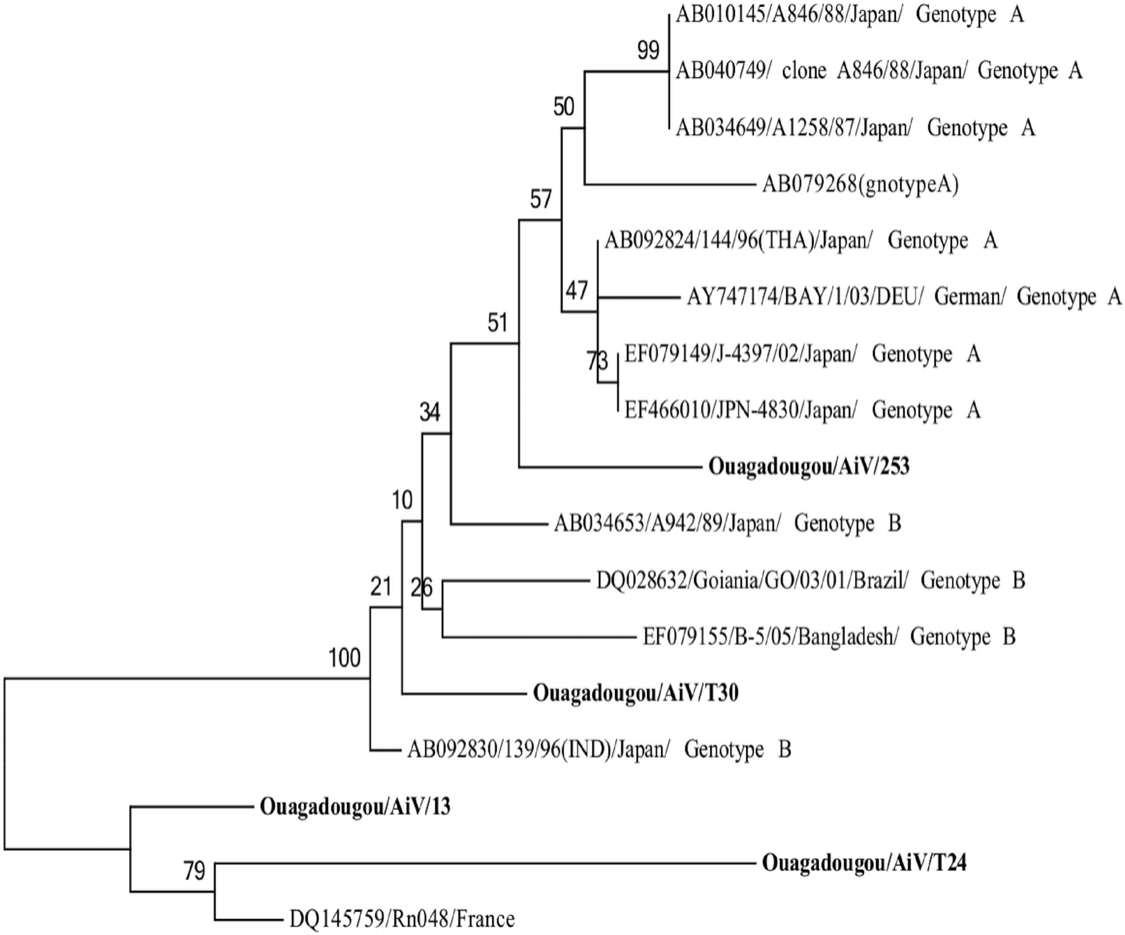
Phylogenetic analysis based on the partial nucleotide sequence (477 bp) of the 3CD-encoding gene of the *Aichivirus A* detected in Ouagadougou, Burkina Faso, between November 2011 and September 2012. The tree was constructed using Maximum Likelihood method. Reference strains of *Aichivirus A* were selected from the GenBank database.

The four *Aichivirus A* strains detected were classified into genotype A (n = 1), genotype B (n = 1) and genotype C (n = 2). Phylogenetic tree based on the partial nucleotide sequence (477 bp) of the 3CD-encoding gene of the *Aichivirus A* is showed in Fig 7. *Aichivirus A* genotype A strain displayed 95% nt (97% aa) with the reference strain J-4397/02/Japan (EF079149); *Aichivirus A* genotype B, 95% nt (98% aa) with the reference strain 139/96(IND)/Japan (AB092830) and *Aichivirus A* genotype C presented 83–97% nt identity (87–94%) with the reference strain RN48/France (DQ145759).

## Discussion

Diarrheal disease is a major cause of worldwide mortality in children. The importance of viruses in children gastroenteritis has been underlined in several reports worldwide and the incidence ranged between 45 and 60% [12–14]. The present study describes detection and characterization of viruses associated with acute diarrhea in Burkinabe children. In our study at least one virus was identified in 85.6% of symptomatic cases, against 72% in the control group. Lower prevalence rates of 35% to 40% have been reported in symptomatic patients in Europe and 60.9% in Gabon [14, 37]. The systematic detection of the five viruses in the current study allowed us to observe a relatively high proportion of co-infection (35.7%) in children with acute diarrhea, which is higher than previously reported in China (5.2%) [38], in America (9%) [39] and in Tunisia (15.2%) [12]. The conditions of the studies, such as the season of sampling, the socioeconomic level of the population, sampling methods and methods of viruses detection, can explain these differences in co-infections rates.

In this study, RVA was the most common virus detected and had the strongest association with disease. This finding was reported in several studies and RVA was mostly associated in severe diarrhea cases and hospitalized young children [37, 40]. The prevalence of RVA in the present study is higher than in a previous study conducted in Burkina Faso, where RVA was detected in 33.8% of the patients [16]. This could be explained by the detection method used in the present study. Indeed, real-time RT-PCR has been demonstrated to be more sensitive than antigen detection by immunochromatographic test [41]. The circulating G and P genotypes showed a diverse pattern, with G9P[8] strains dominating. Previous RVA surveillance studies in Burkina Faso revealed that G1P[8] was the predominant strain from November 2008 to February 2010 [17]. Although being regionally-restricted, these results suggest that the predominant genotype of RVA in Burkina Faso can change rapidly within a short period of time. Others studies have shown in Europe and Asia that large fluctuations in the genotype distribution of human rotaviruses occur continuously from 1 year to another or from one place to another [42, 43]. G9 was detected mostly in combination with P[8], as has been reported elsewhere in the West African sub-region [44, 45]. The unusual G/P type G6P[6] (16.7%), G9P[6] (10.4%) and G12P[8] (6.3%) were also found. Recently, the emergence of G6P[6] in Burkina Faso has been reported [19]. The G6 found in this study related 100% nt (100% aa) identity with RVA/Human-wt/BFA/265-BF/2010/G6P6 (JN116531) previously detected in Burkina Faso and clustered with AF532202/ B1711. These results showed that the same G6 strains continue to circulate in Burkina Faso area. G6 are the most prevalent genotype in cattle worldwide [46], but they are rarely found in humans. However, studies in Gabon and Democratic Republic of Congo showed a high prevalence of genotype G6P[6] among children presenting with diarrhea [14, 47]. Because bovine G6 strains are highly prevalent in cattle, it is possible that the VP7 gene found in this study is derived from a previous transmission event between cattle and humans. G9P[6] rotavirus strains are widespread and have been found in many countries such as Bangladesh, Ukraine, Vietnam and many authors suggested that G9P[6] is a human-animal reassortant [48–50]. Indeed, in the peripheral areas of Ouagadougou, as well as Burkina Faso in general, animals and humans live in close proximity, thus increasing the possibility of RVA transmission between animals and humans. This is the first report of RVA G12P[8] strains in Burkina Faso. These strains have been shown to be emerging in West Africa [51–52] and other continents [53]. G12 strains began to emerge globally, predominantly in combination with either P[6] or P[8] in several countries [53]. The emergence of G12P[8] rotavirus confirms that these strains have the potential to become a sixth common genotype across the world [53, 54].

During this study, 30.7% of RVA was G and P untypable. A low viral load may partially explain this observation. Indeed, a large part of the G and P untypable samples had cycle of quantification values >35 suggesting low viral load. Considering the better sensitivity of real-time RT-PCR used for the detection compared to the end-point RT-PCR used for genotyping, RVA can be detected but not genotyped in these low viral load samples. Another possible explanation is that untypable strains have accumulated point mutations not recognized by the primers used. Finally, the presence of RVA genotypes not yet recognized could not be excluded. Great variability in circulating rotavirus has been observed in African countries from year to year [54] and many rotavirus strains from sub-Saharan Africa remain untypable.

NoV is a causative virus of acute gastroenteritis in children and has been known to contaminate food causing viral outbreaks affecting people [55]. In this study, NoV-GII was detected in 18.3% and NoV-GI in 2.7% cases of children with diarrhea. The higher prevalence of NoV GII over GI in acute gastroenteritis cases has been reported from other parts of the world [56, 57]. Prevalence of NoV-GII found in this study is higher than that reported in Gabon, China and Paraguay which found 13.9%, 27.2% and 58% respectively [14, 38, 58].

The presence of NoV-GII was 3.5 times higher in the symptomatic group than in the control group. In our study, NoV-GII was one of the major viruses associated with diarrhea (*p* = 0.04) unlike NoV-GI (*p* = 0.5). This study supports the increasing recognition that NoV-GII is commonly responsible for sporadic cases of gastroenteritis in children [10]. Following sequence analysis, six genotypes of genogroup II were found in both symptomatic and asymptomatic patients. NoV-GII.4 was the most prevalent genotype detected during the whole study, unlike a previous study on NoV (22.2%) in another geographical region of Burkina Faso [21]. This genotype is considered as the predominant genotype responsible for the majority of outbreaks of gastroenteritis worldwide [10]. During this study, the NoV-GII.4 variant 2012 (Sydney) was frequently detected for the capsid. This GII.4 variant emerged and caused the majority of the acute gastroenteritis outbreaks worldwide [59–62]. However the NoV-GII.4 were variant 2004 (Hunter) for the RNA polymerase. This result suggests the possibility of recombination occurred in the open reading frame (ORF)1/ORF2 overlap [63].

The presence of adenovirus in the symptomatic group was significantly lower than in the control group (p = 0.01). However, in this study, adenovirus types generally associated with diarrhea were minor. Indeed, the molecular characterization of adenovirus revealed the presence of only 12 (20.3%) strains of adenovirus generally associated with diarrhea (i.e. types 40, 41 and 31) [64, 65]. This finding is higher compared to a study in Republic Democratic of Congo, which found 5.5% of adenovirus (type 40/41) [66]. Others AdV genotypes found in this study are generally known causes of acute respiratory disease or keratoconjunctivitis in children [67].

There is no significant difference between the prevalence of SaV, AstV and AiV in the symptomatic and asymptomatic population. The lack of clinical history of patient was a limit of this study. However, other studies suggesting that these viruses cause asymptomatic infections or mild forms of gastroenteritis which are treated at home [68, 69].

SaV was detected in 10.3% cases of children with diarrhea. This result was consistent with those of published reports that showed that its prevalence is usually much lower than norovirus [69, 70]. Genogroup II SaV (87.5%) was found to be the most common genogroup, with GII.1 the most predominant strain. GII was the predominant sapovirus strain worldwide [71–73]. One sapovirus found in this study showed greatest sequence homologies with Sakaeo-15 (Accession number AY646855), which represents a novel GII genetic cluster [74].

AstV was found in 4.9% of children with diarrhea. This prevalence is similar to that reported in seven provinces of China [75], Germany [69] and Ivory Coast [76], which found 5.5%, 4% and 4% prevalence, respectively. In contrast, one study in Gambia and Kenya reported AstV in 9.9% of all stools collected [77]. AstV type 1 (41.7%) was the most predominant strain and Burkinabe strains presented 95.2–100% aa identity with the AstV-1 reference strain (Accession number L23513, Oxford). This human AstV type is the most common worldwide [78–80]. Uncommon (type 2 and 5) and rare (type 8) serotypes were also found during this study. Serotype 8 may represent an emerging AstV type worldwide [80, 81].

Only four AiV A were found in this study and belonged to genotype A, B and C, suggesting that this virus is rare in children with diarrhea in Ouagadougou, Burkina Faso. *Aichivirus A* has been found at low incidence in patients with gastroenteritis in several regions around the world, including Europe [82] and Africa [83].

## Conclusion

A limitation of this study is the small size of control group (n = 50). Furthermore, due to the large number of untypable strains found in this study, the genetic diversity of enteric viruses in Burkinabe children may have been underestimated.

Documenting the etiology of diarrhea is important to guide vaccine development, design prevention and treatment strategies. In this study, RVA and NoV-GII were the two predominant viruses associated with diarrhea. The introduction of affordable viral diagnosis in our pediatric hospitals will improve patient care by reducing the unnecessary use of antibiotics. The relative high incidence of infections reported here suggests a possible problem of hygiene, such as water contamination, or sociodemographic level of population.

Molecular characterization reported a great diversity of strains. Thus, information about strain diversity and emerging strains would also be useful for better management of preventive strategies such as vaccination. These results highlight the importance of continuous surveillance of gastroenteritis viruses in Burkina Faso.
